# AMF inhibit the production of phenolic acid autotoxins at the seed-filling stage in soybeans with continuous monocropping

**DOI:** 10.1186/s12870-024-05330-y

**Published:** 2024-07-31

**Authors:** Hao Shi, Chengcheng Lu, Yunshu Wu, Lei Wang, Baiyan Cai

**Affiliations:** Engineering Research Center of Agricultural Microbiology Technology, Ministry of Education & Heilongjiang Provincial Key Laboratory of Ecological Restoration and Resource Utilization for Cold Region & Key Laboratory of Molecular Biology, College of Heilongjiang Province, School of Life Sciences, Heilongjiang University, Harbin, 150080 China

**Keywords:** Soybean continuous monocropping, Autotoxicity disorder, Phenolic acids, AMF, *F. mosseae*

## Abstract

**Background:**

Soybean is the main oil crop in Northeast China. Continuous monocropping is more commonly used for soybean production due to rising market demand and arable land constraints. However, autotoxic substances, such as phenolic acids, produced by continuously cropped soybean can reduce yield and quality. The mycorrhiza formed of Arbuscular mycorrhizal fungi (AMF) and plant roots regulate the metabolic activities of the host plant and increase its disease resistance. The main purpose of this study was to inhibit the production of phenolic acids and determine the adverse effects on the growth of continuous monocropping soybean by inoculating *Funneliformis mosseae* (*F. mosseae*).

**Results:**

Transcriptomics results showed that the production of phenolic acids in continuous monocropping soybean roots was mainly regulated by the expression of the *CHS6*, *PCL1*, *SAMT*, *SRG1*, and *ACO1* genes, and the expression of these genes was significantly downregulated after inoculation with *F. mosseae*. Metabolomics results showed that continuous monocropping soybean roots inoculated with *F. mosseae* inhibited phenolic acid production through the phenylpropane biosynthetic, α-linoleic acid, linoleic acid, and other metabolic pathways. Phenolic acids in the phenylpropane metabolic pathway, such as 4-hydroxybenzoic acid, phthalic acid, and vanillic acid, decreased significantly after inoculation with *F. mosseae*. The combined analysis of the two showed that genes such as *YLS9* and *ARF3* were positively correlated with 4-hydroxybenzoic acid and so on, while genes such as *CHS6* and *SRG1* were negatively correlated with butyric acid and so on.

**Conclusion:**

*F. mosseae* regulated the expression of functional genes and related phenolic acid metabolic pathways produced by continuous monocropping soybean roots, inhibiting the production of phenolic acid autotoxic substances in continuous cropped soybean, and slowing down the disturbance of continuous monocropping. This study provides a new solution for continuous monocropping of plants to overcome the autotoxicity barrier and provides a new basis for the development and utilization of AMF as a biological agent.

## Background

Soybean provides human food and animal feed as a globally consumed agricultural product, It also provides important feedstock, such as edible oil and biodiesel [1]. Soybean is an important crop in Northeast China, particularly in Heilongjiang Province, accounting for one-third of total national production [2]. Due to the worldwide increasing demand for soybeans and market regulation, the soybean planting area is expanding rapidly, and continuous cropping is a common way to grow soybeans. Soybean being planted continuously can lead to plant stunting, increased pests and diseases, resulting in a decrease in seed quality and yield, and a reduction in the economic benefits of soybeans [3], for example, compared to conventional crop rotation, Xu et al. [4] concluded that soybeans with continuous cropping reduced yields by 18.6% and 35.4% in 1 and 2 years, respectively. Continuous cropping of soybean leads to excessive secretion of chemosensory substances, such as organic acids, by the root system, which causes autotoxicity in soybean plants [5]. Phenolic acids are an important secretion of the root system of continuously cropped soybeans, which are involved in chemosensory effects. Phenolic acids include coumaric acid, mustard acid, vanillic acid, ferulic acid, p-hydroxybenzoic acid, and phthalic acid [6]. Phenolic acids affect nutrient absorption, inhibit photosynthesis, disrupt dark respiration and ATP synthesis, affect cell division, and inhibit plant growth [7, 8]. Previous studies have shown that phenolic acids have a significant inhibitory effect on the photosynthetic activity of European and American poplar, which is due to their negative effect on the activity of a nitrogen assimilation enzyme, thus reducing the amount of protein and amino acid converted into protein [9]. Vanillic acid, which is found in soybean root exudates, has a considerable effect on the rhizosphere microbial community [10]. Phenolic acids induce growth and toxin production in soil-borne pathogenic fungi, e.g., inter-root phenolic acids of *Rehmannia glutinosa* induce mycelial growth and the production of toxin by the pathogenic fungus *Fusarium oxysporum* [6]. As a result, the soil ecology under continuously cropped conditions severely affects plant growth, creating an autotoxic effect, and the plant produces a barrier to continuous cropping. Therefore, the main objective of this study was to slow down the barriers to the production of phenolic acid autotoxins in continuous cropped soybeans by inoculation with AMF mycorrhizal agents.

AMF are ubiquitous soil microorganisms that form symbiotic relationships with more than 80% of terrestrial plants. AMF provide a direct physical relationship between the soil and plant roots, significantly improve the absorption and utilization of nutrients by host plants, and improve plant tolerance to biotic and abiotic stressors [11–13]. AMF can improve plant nutrition and health and promote plant growth [14]. Inoculation with AMF increased biomass accumulation in tomato [15] and also reduced the negative effects of thallium stress on mineral element uptake and growth in soybean [16]. This is partly because mycorrhizal symbionts help the host plant to absorb water and nutrients [17], and partly because inoculation with AMF promotes chlorophyll synthesis in the host plant, increasing root surface area and root vigour, which in turn promotes plant growth [18]. AMF are able to influence the activity of secondary metabolism in host plant cells. Studies have shown that inoculation of AMF can effectively reduce the accumulation of phenolic acids in plant roots and soil after continuous cropping of citrus [19] and watermelon [20] under greenhouse potting conditions. Therefore, it is important to study the relationship between AMF and secondary metabolism in plants.

This study used a combined transcriptomics and metabolomics analysis to investigate the effect of inoculation with AMF on the production of phenolic acid self-toxic metabolites in the root system of continuous soybean, and hypothesised that the inoculation with AMF could inhibit the expression of genes regulating phenolic acids in the root system of continuous soybean, modulate the enrichment of metabolic pathways, and reduce the production of phenolic acids in the root system, which would ultimately alleviate the continuous cropping disorder in soybean. The main objective of this study is to elucidate the molecular mechanism of AMF to inhibit the production of phenolic acid autotoxic substances in continuous soybean, and to provide a theoretical basis for the application of AMF in agricultural production and improvement of the continuous cropping disorder, so as to promote the sustainable development of agriculture.

## Methods

### Test materials

The experiment was conducted from May to September 2022 at the Experimental Station of the Sugar Research Institute of the Harbin Institute of Technology (46°40′N, 130°10′E). The test soils were selected from soils that had not been planted with soybean (previous crop was maize) and soybean soils that had been cropped for 3 years (soybean had been planted for 2 consecutive years), and the background values of the soils are shown in Table 1. The soybean variety was Heinong 88, which is a widely grown high-protein variety in Heilongjiang Province. The AMF agent was *F. mosseae*, and the original strain of *F. mosseae* was purchased from the Institute of Plant Nutrition and Resources, Beijing Academy of Agricultural and Forestry Sciences, and contained approximately 20 spores/g of spore inoculum.

**Table 1:** Background values for the test soils

	Organic matter (g/kg)	Total nitrogen (g/kg)	Total potassium (g/kg)	Total phosphorus (g/kg)	Alkali hydrolyzed nitrogen (mg/kg)	Available phosphorous (mg/kg)	Available potassium (mg/kg)	Effective sulfur (mg/kg)
0 year of continuous monocropping	26.22	1.63	25.41	5.24	129.13	13.27	207.27	15.84
3 years of continuous monocropping	26.13	1.54	24.83	4.82	127.90	12.88	204.33	12.96

### Experimental design

The experiment was conducted in pots by digging 0–20 cm field tillage soil from soybean soil of years 0 and 3 of continuous monocropping, passing it through a 1 cm sieve, and air-drying it for use. Each pot was filled with 2.5 kg of air-dried soil. Five soybean seeds were sown in each pot and three healthy seedlings of uniform growth were retained after the soybeans emerged.

The experiment was set up with four treatments: (1) Year 0 soil not inoculated with *F. mosseae* (25 g of autoclaved *F. mosseae* mycorrhizal agent mixed with 2.5 kg of soil at the time of sowing was used as a control), labeled C; (2) year 0 soil inoculated with *F. mosseae* (25 g of *F. mosseae* mycorrhizal agent mixed with 2.5 kg of soil at the time of sowing), labeled T; (3) soil not inoculated with *F. mosseae* for 3 years of continuous monocropping (25 g of autoclaved *F. mosseae* mycorrhizal agent mixed with 2.5 kg of soil at the time of sowing), labeled as CC; (4) soil inoculated with *F. mossea*e (25 g of *F. mosseae* mycorrhizal agent mixed with 2.5 kg of soil at the time of sowing) for 3 years of continuous monocropping, and labeled CT.

Each treatment in the experiment was subjected to three biological replications, with 15 pots were planted per treatment, for a total of 60 pots, and sown in mid-May 2022, with the pots and pans randomly arranged in a field and regularly switched to prevent marginal effects. The soybean plants were watered and weeded normally during the reproductive period, and the potting soil was kept moist and free of other weed growth, and samples were taken at 30, 45, 60, 75 and 90 d after soybean emergence, during which the soybean root system was rinsed quickly and repeatedly with distilled water to remove surface soil, and three biological replicates were collected from each treatment for the determination of physiological indices. Soybean roots were taken at the time of highest *F. mosseae* colonization rate (75 d after emergence), quickly frozen in liquid nitrogen and stored in a – 80℃ refrigerator, and retained for transcriptomics and metabolomics analyses.

### Test methods

#### Determining the soybean growth indicators and the AMF colonization rate

Determination of plant height [21]: Three randomly selected soybean plants in the same treatment were selected, the soil attached to the root system was shaken off, and the height of the plant from the cotyledon scar to the top of the plant was measured with a ruler.

Determination of fresh weight and dry weight [22]: The soybean plants were cut (above and below ground parts were labeled separately), weighed, and recorded, then placed in an oven at 80 °C for 30 min, which was adjusted to 70 °C to dry to a constant weight and the material was accurately weighed and recorded.

AMF colonization rate: Starting from the growth of soybean to 30 d, the mycorrhizal colonization rate was determined by examining the root system at 7 d intervals. Referring to the acidic magenta staining method of Phillips [23], the fibrous roots of the soybean were washed and cut into 1 cm segments, stained, decolored, and sectioned, and mycorrhizal colonization was observed under a light microscope. Mycorrhizal colonization rate = (number of infested root segments/total number of root segments observed) × 100%.

### Extraction of metabolites and the analytical methods for soybean roots

Roots were collected from soybean at the seed-filling stage, and soybean roots were rinsed with distilled water to remove surface soil, and three biological replicates were collected for each treatment. Soybean root samples were vacuum freeze-dried, ground, and pulverized in a grinder at 30 Hz for 1.5 min. The samples were ground into a powder, 100 mg was weighed and added to an aqueous solution containing 70% methanol from the internal standard, shaken for 30 s, and centrifuged (4 °C, 10,000 g) for 10 min. The supernatant was filtered, and the same volume of all samples to be tested was mixed as a quality control sample to assess the reproducibility of the results.

The extracted compounds were analyzed by a liquid chromatography-electrospray-tandem mass spectrometry (LC-ESI-MS/MS) system (SCAA-104, 0.22 μm pore size, Amperex, Shanghai, China). A 2 µL aliquot of sample was injected into a Waters ACQUITY UPLC HSS T3 C18 column (2.1 mm × 100 mm, 1.8 μm). The effluent from the chromatographic column was fed into the ESI-QTRAP-MS for mass spectrometry analysis. LIT and triple quadrupole scans were performed on a triple quadrupole linear ion trap mass spectrometer (QTRAP) AB Sciex QTRAP 6500 system, operating in positive ion mode and controlled by Analyst 1.6.1 software (AB Sciex). The instrument operating parameters were set and the monitoring mode was set to multi-response monitoring.

### Methods for analyzing the soybean root transcriptome sequence

Soybean roots were collected during the seed-filling stage of soybean, and the roots were rinsed quickly and repeatedly with distilled water to remove the soil on the surface, and then, the surface of the roots was blotted dry with absorbent paper. The 1 g samples were accurately weighed and frozen with liquid nitrogen. Three biological replicates were collected for each treatment.

Total RNA was extracted using Trizol reagent kit (Invitrogen, Carlsbad, CA, USA) according to the manufacturer’s protocol. RNA quality was assessed on an Agilent 2100 Bioanalyzer (Agilent Technologies, Palo Alto, CA, USA) and checked using RNase free agarose gel electrophoresis. After total RNA was extracted, eukaryotic mRNA was enriched by Oligo(dT) beads, while prokaryotic mRNA was enriched by removing rRNA by Ribo-Zero™ Magnetic Kit (Epicentre, Madison, WI, USA). Then the enriched mRNA was fragmented into short fragments using fragmentation buffer and reverse transcripted into cDNA with random primers. Second-strand cDNA were synthesized by DNA polymerase I, RNase H, dNTP and buffer. Then the cDNA fragments were purified with QiaQuick PCR extraction kit(Qiagen, Venlo, The Netherlands), end repaired, poly(A) added, and ligated to Illumina sequencing adapters. The ligation products were size selected by agarose gel electrophoresis, PCR amplified, and sequenced using Illumina HiSeq2500 by Gene Denovo Biotechnology Co. (Guangzhou, China).

A variety of algorithmic models for calculating differentially expressed genes are provided in the edge R package (https://www.r-bloggers.com/its-easy-to-cite-and-reference-r/), which was used to analyze differential gene expression changes between groups. Differential expression fold change (log_2_ fold change) indicates the ratio of expression between two groups of samples, therefore, differentially expressed genes (DEGs) were screened with *p* < 0.05, |log_2_FC| > 1. DEGs were annotated and subjected to GO functional enrichment and KEGG pathway analyses using the Mercator web tool.

### Statistical analysis

Data are expressed as mean ± SD of three independent biological replicates. Data were analyzed using Excel 2019 (Microsoft Inc., Redmond, WA, USA) and SPSS 26.0 (IBM Corp., Armonk, NY, USA) software. Significant differences were detected by two-way ANOVA and statistical differences with a *p* value < 0.05 were considered significant. Graphs were plotted using Origin 2023 software. Combined analysis of transcriptomic and metabolomic data and correlation analyses were performed using R 4.1.0 software.

## Results

### Changes in mycorrhizal colonization rates during the soybean reproductive period

**Fig. 1:**
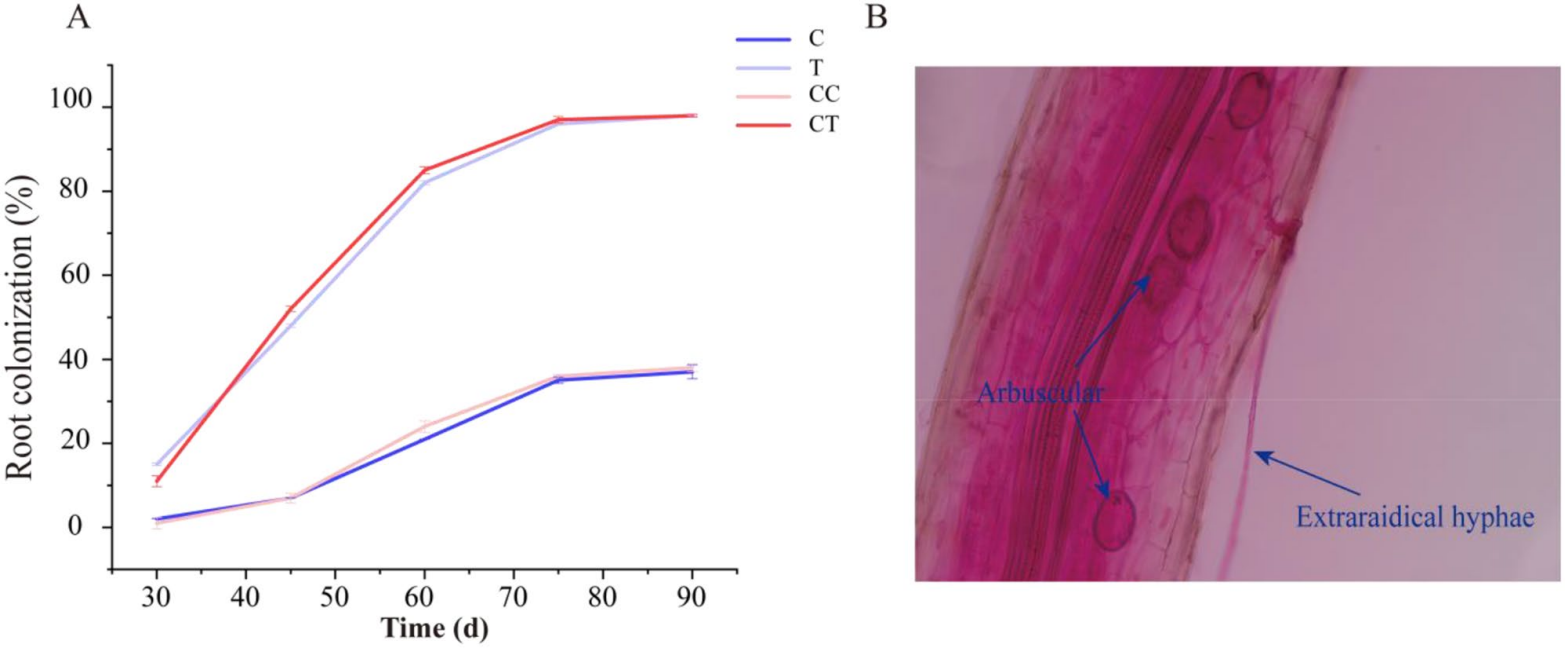
Trends in the mycorrhizal colonization rate and morphological characteristics of colonization in soybean. **A**, Changes in soybean mycorrhizal colonization rate. **B**, Morphological characteristics of soybean mycorrhiza. (C: control; T: 0 years of continuous monocropping + AMF; CC: 3 years of continuous monocropping; CT: 3 years of continuous monocropping + AMF)

As shown in Fig. 1A, the colonization rate of each treatment group increased gradually with the growth and development of the soybean plants during the reproductive period, and the colonization rate peaked and gradually leveled off when the plants were 75 d old. The colonization rate of each treatment group increased with the growth and development of the soybean during the reproductive period. Groups T and CT had a colonization rate of about 98%, whereas the colonization in groups C and CC was mainly due to colonization of the root system by the pre-existing AMF in the soil, with a root colonization rate of nearly 40%. A good symbiotic relationship was observed between *F. mosseae* and the soybean root system 75 d after inoculation, when the mycorrhizal structure gradually grew and a large number of hyphae and vesicles appeared (Fig. 1B).

### Changes in soybean fertility growth indicators

**Fig. 2:**
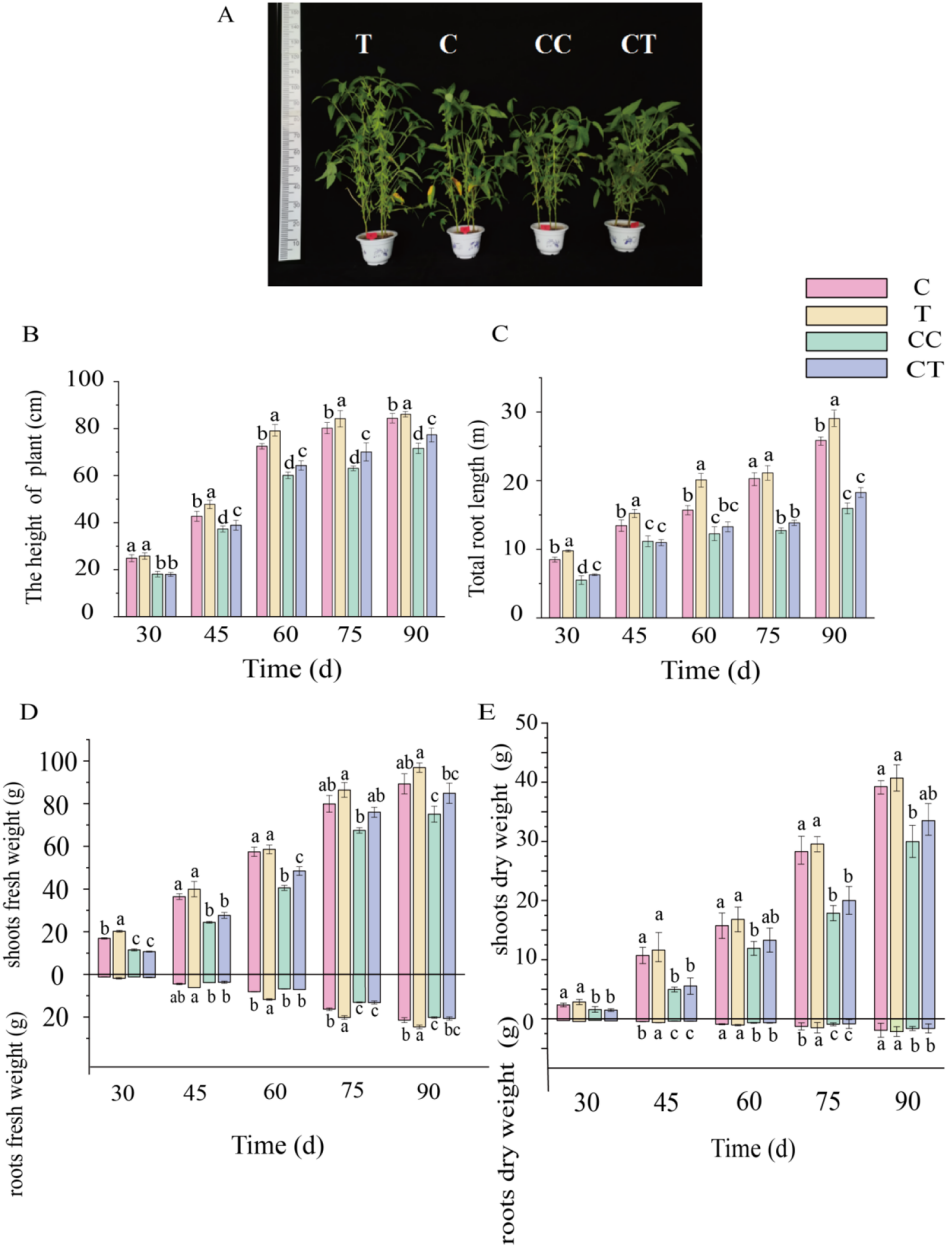
Changes in plant growth indices during the soybean fertility period. **A**, 75 d growth of soybean. **B**, changes in plantthe height of plant. **C**, changes in total root length. **D**, changes in fresh weight above and below groundshoots and roots. **E**, changes in dry weight above and below groundshoots and roots. (C: control; T: 0 years of continuous monocropping + AMF; CC: 3 years of continuous monocropping; CT: 3 years of continuous monocropping + AMF)

As shown by the growth of soybean in Fig. 2A, continuous monocropping affected the growth of soybeans, and a series of symptoms, such as yellowing of leaves and dwarfing appeared in the plants after 3 years of continuous monocropping, suggesting that obstacles to continuous monocropping promoted the production of autotoxic substances by the soybeans and the plants are in a state of stress, leading to malnutrition and stunted growth and development.

Changes in growth indicators during the soybean reproductive period are shown in Fig. 2B, 2 C, 2D, 2E. The differences in plant height, root length, above-ground and below-ground fresh weight, and above-ground and below-ground dry weight of soybean were significant (*p* < 0.05) when group C was compared with group CC. Continuous monocropping significantly inhibited the growth of soybeans, indicating that continuous monocropping of soybeans produces growth barriers. The F treatment promoted the growth of soybean in group C compared with group F, resulting in a significant (*p* < 0.05) increase in plant height, root length, and aboveground and belowground fresh and dry weights, suggesting that inoculation with *F. mosseae* promotes the soybean growth indices. The CT treatment promoted an increase in soybean plant height, root length, above-ground and below-ground fresh weight, and above-ground and below-ground dry weight compared with the CC and CT groups, and the differences between treatments were significant (*p* < 0.05), indicating that inoculating continuous soybeans with *F. mosseae* significantly improved the growth indices and that *F. mosseae* inhibits continuously cropped soybean.

### Annotation analysis of functional genes regulating phenolic acid production in soybean roots

**Fig. 3:**
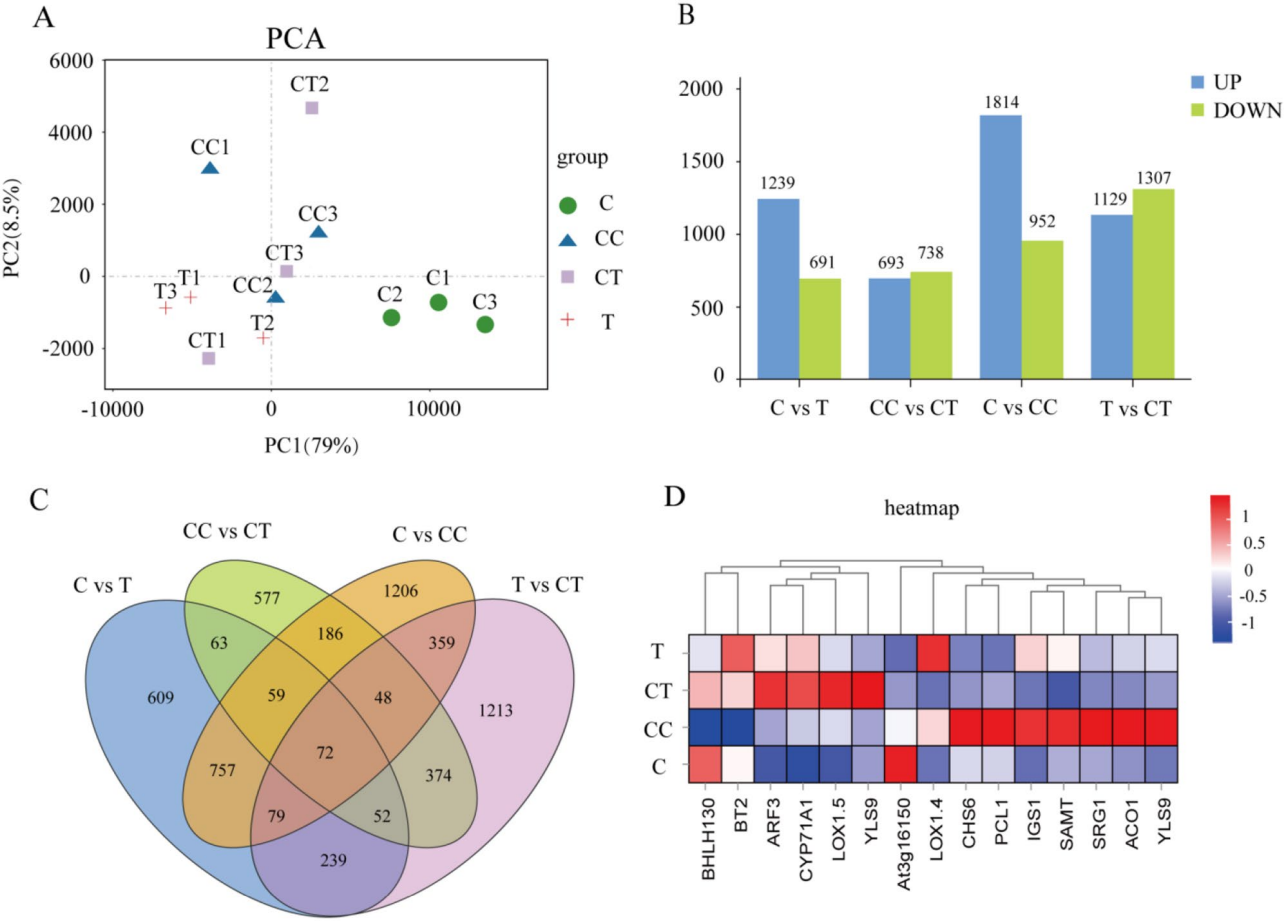
Analysis of the differentially expressed genes in the four treatment groups. **A**, PCA analysis. **B**, bar chart for analysis of the number of DEGs between treatment groups. **C**, Wayne’s plot of the DEGs between treatment groups. **D**, heatmap of target DEGs between the treatment groups. (C: control; T: 0 years of continuous monocropping + AMF; CC: 3 years of continuous monocropping; CT: 3 years of continuous monocropping + AMF). (*Figure note*: red indicates an increase in gene expression, blue indicates a decrease in gene expression.)

To assess the reproducibility of the soybean DEGs library, the transcriptome profiles of the 12 analyzed samples were subjected to principal component analysis (Fig. 3A). Dispersion of the components on PC1 was 79%, and the samples had good reproducibility. Statistical methods were used to analyze the DEGs between treatments by comparing the RNA-seq data between treatment groups with log_2_ fold change (FC) > 1 and *p* < 0.05 thresholds as criteria for identifying the DEGs. As shown in Fig. 3B, the C vs. T group had a total of 1,930 DEGs, of which 1,239 were upregulated and 691 were downregulated; the CC vs. CT group had a total of 1,431 DEGs, of which 693 were upregulated and 738 were downregulated; the C vs. CC group had a total of 2,766 DEGs, of which 1,814 were upregulated and 952 were downregulated, and the T vs. CT group had a total of 2,436 DEGs, of which 1,129 were upregulated and 1,307 were downregulated.

**Fig. 4:**
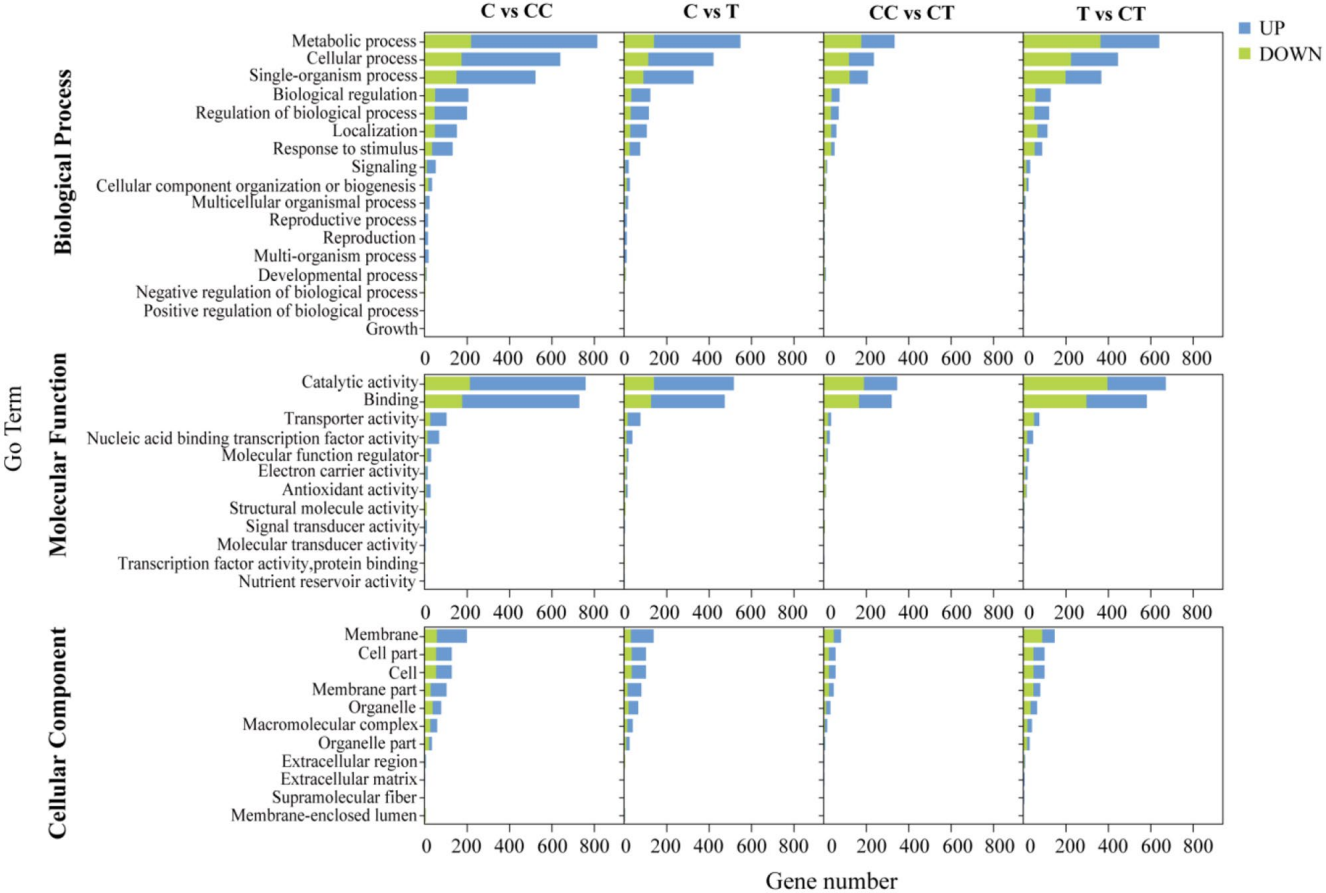
Secondary bar chart of GO enrichment classification of the DEGs in the four groups: C vs. CC, CC vs. CT, C vs. T, and T vs. CT. (C: control; T: 0 years of continuous monocropping + AMF; CC: 3 years of continuous monocropping; CT: 3 years of continuous monocropping + AMF). (*Figure note*: From top to bottom, the three boxes indicate metabolic bioprocesses (BP), molecular functions (MF), and cellular components (CC), respectively. *Figure note*: Blue indicates upregulation of expression and green indicates downregulation of expression.)

According to Fig. 3 C, there were a total of 365 gene expression comparisons in the C vs. CC and CC vs. CT groups. These 365 expressed genes were mainly associated with the two factors of continuous monocropping and inoculation with *F. mosseae*, and the 365 DEGs contained target genes for phenolic acids produced by continuous monocropping of soybean, so a heatmap analysis of the target genes was performed (Fig. 3D). To further understand the function of the DEGs and their involvement in relevant biological processes, GO enrichment analyses were conducted. The DEGs from the four groups were significantly enriched in GO subclasses involving biological processes, molecular functions, and cellular components (Fig. 4), and the enriched GO terms were metabolic processes, catalytic activities, and cellular membranes.

The DEGs significantly enriched in the CC-treated group were *CHS6*, *PCL1*, *SAMT*, *SRG1*, and *ACO1*. The DEGs significantly enriched in the CT group were *BHLH130*, *ARF3*, *CYP71A1*, *LOX1.5*, and *YLS9*. In the C vs. T comparison, the T treatment significantly enriched DEGs for *LOX1.4*. In the T vs. CT comparison, the CT treatment significantly enriched DEGs for *LOX1.5*, and *YLS9*.

**Fig. 5:**
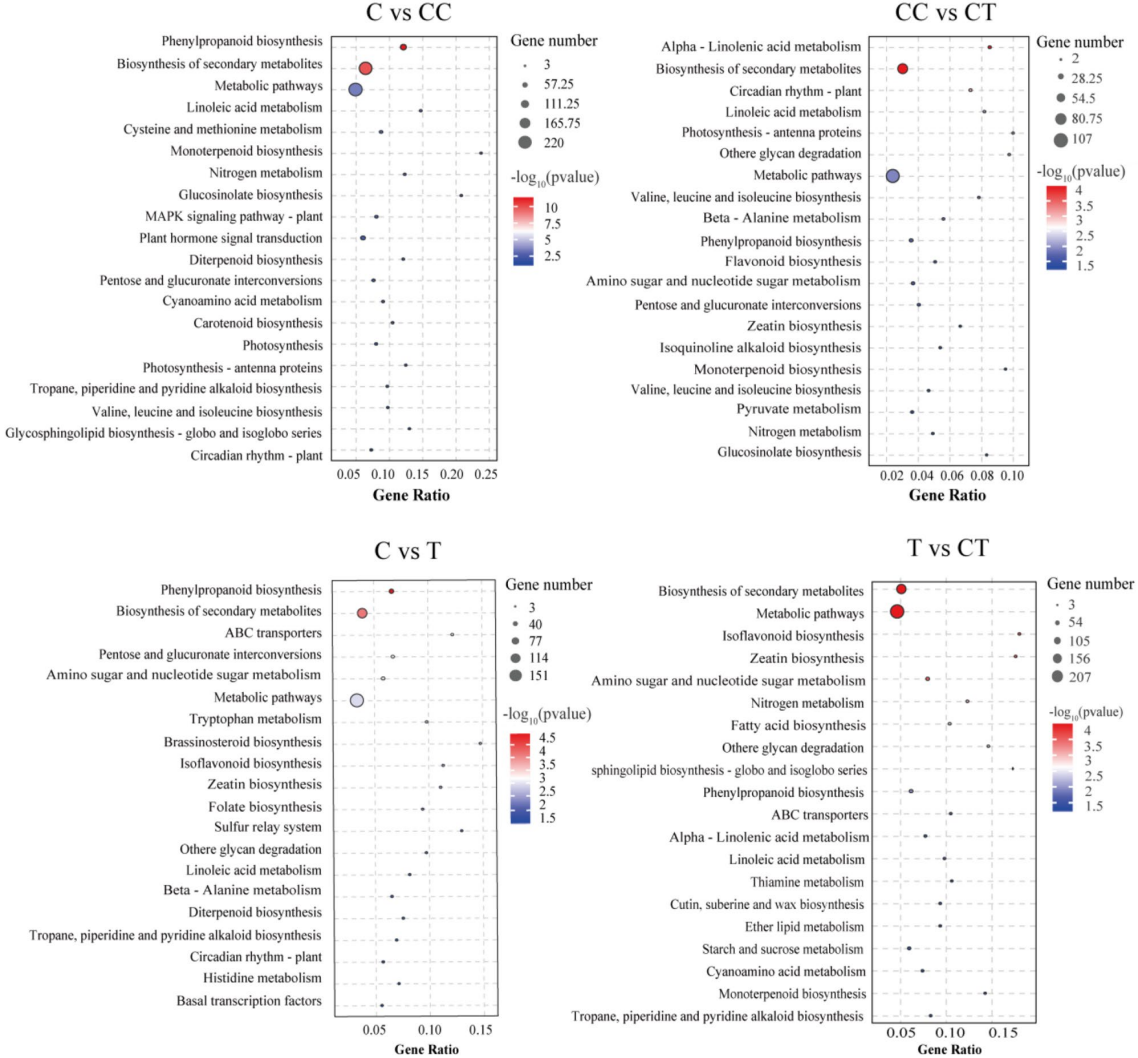
KEGG-enriched bubble plots of the DEGs in the four groups of C vs. CC, CC vs. CT, C vs. T, and T vs. CT. (C: control; T: 0 years of continuous monocropping + AMF; CC: 3 years of continuous monocropping; CT: 3 years of continuous monocropping + AMF). (*Figure note*: The size of the bubbles indicates the number of enriched DEGs, with larger bubbles indicating more DEGs enriched in the pathway; the color of the bubbles indicates the − log_10_(*p*) value, with a redder color indicating a more significant correlation of the values.)

KEGG enrichment analyses were performed to further identify the specific metabolic pathways in which these DEGs are involved, and the results are shown in Fig. 5. The C vs. CC analyses showed that pathways, such as phenylpropane biosynthesis and cysteine and methionine metabolism were significantly enriched in the CC-treated group. Phenolic acids produced by the plants were mainly synthesized through the phenylpropane biosynthetic pathway, which was significantly enriched in the CC treatment group, suggesting that phenolic acids are produced by the continuously cropped roots of soybeans. The CC vs. CT analysis showed significant enrichment of α-linoleic acid metabolism and linoleic acid metabolism pathways in the CT group. Linoleic acid metabolism plays a key role in the regulation of the immune response, cell membrane structure and function, regulation of photosynthesis, and stress response. Enrichment of the linoleic acid metabolic pathway in the CT-treated group suggests that inoculation with *F. mosseae* enhances linoleic acid metabolism in plants and increases resistance to stress. The C vs. T comparison showed that pathways, such as ABC transporter and interconversion of pentose and glucuronic acid, were significantly enriched in group T. The ABC transporter has important physiological functions in the transport of cytotoxins and immunomodulation, and glucuronic acid binds to a variety of hazardous substances with detoxifying effects. The T vs. CT comparison showed that the CT group was significantly enriched in isoflavone biosynthesis and other pathways, and that isoflavones are beneficial for legumes in the responses to biotic and abiotic stressors and to induce the formation of root nodules.

### Analysis of phenolic acids and their metabolic pathways in the soybean root system

A total of 661 metabolites containing 56 classes, such as lipids (33), benzene and substituted derivatives (25), flavonoids (18), carboxylic acids and their derivatives (17), organic acids (17), coumarins and their derivatives (12), and amino acids and their derivatives (12) were detected in soybean root secretions (Table 2).

**Table 2:** Metabolite identification results

Type	Number	Percentage (%)
Total	661	100
Unknown	427	64.599
Lipids	33	4.992
Benzene and substituted derivatives	25	3.782
Isoflavones	20	3.062
Flavonoids	18	2.723
Carboxylic acids and their derivatives	17	2.572
Organic acid	17	2.572
Coumarin and its derivatives	12	1.815
Amino acid derivatives	12	1.815
Indoles and derivatives	5	0.756
Cinnamaldehyde	4	0.605
1,3-diarylpropionic acid	3	0.454
Organic nitrogen compound	3	0.454
Fern compounds and their derivatives	3	0.454
Steroids and steroid derivatives	3	0.454
5’-Deoxyribonucleic acid	2	0.303
Carbohydrates and conjugates	2	0.303
Others	55	8.285

**Fig. 6:**
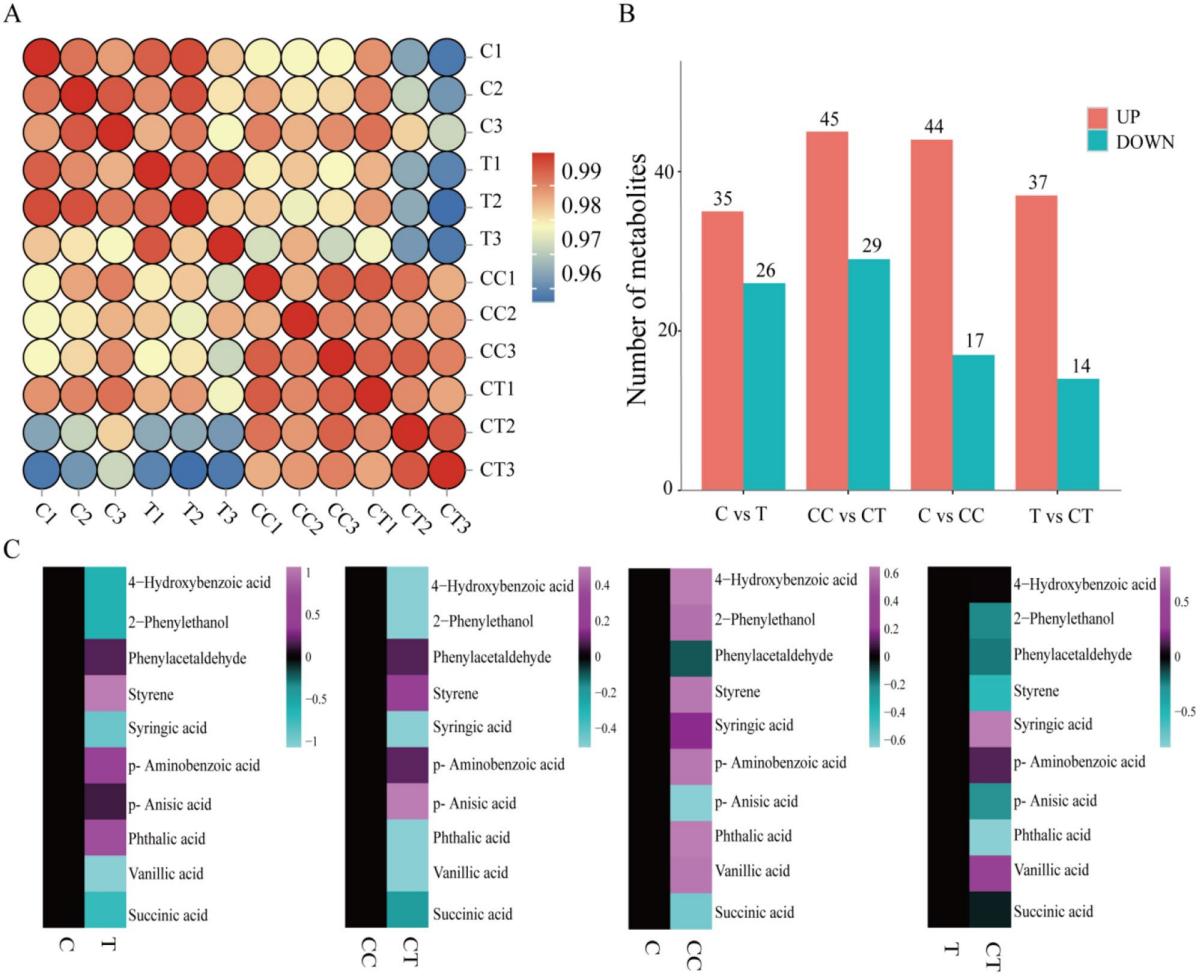
Analysis of differentially expressed metabolites among the four treatment groups.**A**, Heatmap of the relative abundance of metabolites in the four treatments (*Figure note*: red indicates an increase in the content of the substance, blue indicates a decrease in the content of the substance). **B**, Number of differentially expressed metabolites in the four groups of C vs. CC, CC vs. CT, C vs. T, and T vs. CT (*Figure note*: red indicates an increase in the differentially expressed metabolite, and green indicates a decrease in the differentially expressed metabolite). **C**, Change in the relative content of the target phenolic acids in the four groups of C vs. CC, CC vs. CT, C vs. T, and T vs. CT. (*Figure note*: purple indicates upregulation of differentially expressed metabolites, green indicates downregulation of differentially expressed metabolites.). (C: control; T: 0 years of continuous monocropping + AMF; CC: 3 years of continuous monocropping; CT: 3 years of continuous monocropping + AMF).

The relative content of phenolic acids in the root system differed among the four treatment groups (Fig. 6A). Further screening of the DAMs by applying thresholds (VIP ≥ 1, *t*-test *p* value < 0.05) (Fig. 6B) revealed 61 differentially expressed metabolites in the C vs. CC group, 61 differentially expressed metabolites in the C vs. T group; 74 differentially expressed metabolites in the CC vs. CT group, and 51 differentially expressed metabolites in the T vs. CT group.

The development of succession disorder in plants is mainly due to phenolic acids secreted by the root system. In this study, 10 phenolic acids from 25 benzene and substituted derivatives were centrally analyzed (Fig. 6 C), of which 4-hydroxybenzoic acid, phenylethanol, butyric acid, p-methoxybenzoic acid, phthalic acid, and vanillic acid increased significantly after continuous monocropping of soybeans, whereas these phenolic acids were significantly lower after inoculation with *F. mosseae*, suggesting that inoculation with the AMF reduces the phenolic acids in continuously cropped soybeans.

**Fig. 7:**
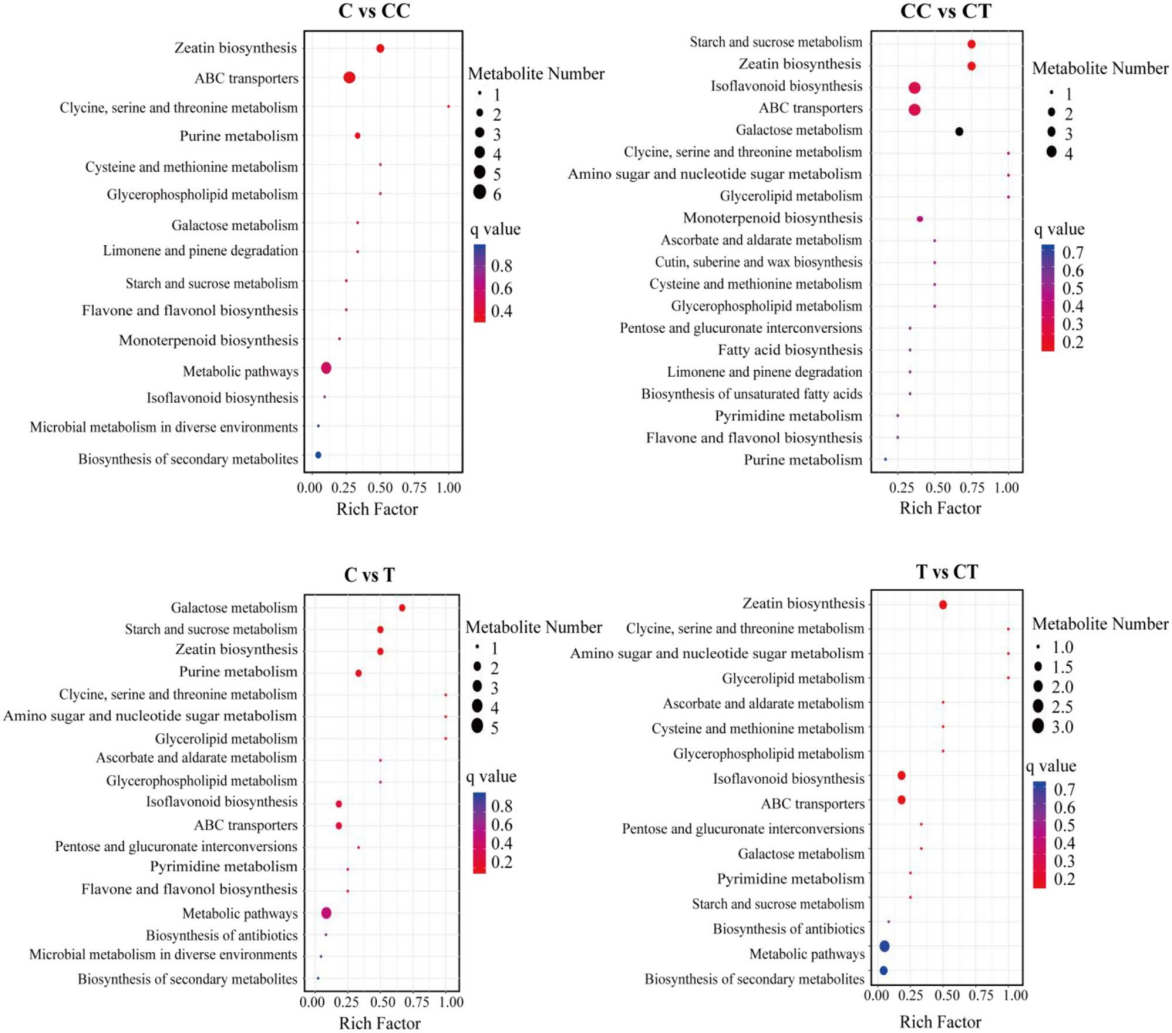
Bubble plots of the top 20 KEGG-enriched pathways of metabolic pathways in the four comparison groups of C vs. CC, CC vs. CT, C vs. T, and T vs. CT. (C: control; T: 0 years of continuous monocropping + AMF; CC: 3 years of continuous monocropping; CT: 3 years of continuous monocropping + AMF). (*Figure note*: The size of the bubbles indicates the number of enriched metabolic pathways, while the color of the bubbles indicates the size of the *q*-value.)

Analysis of the signaling pathways for differentially accumulating metabolites reveals the major biochemical and signaling pathways involved in the differential accumulation of these metabolites. Therefore, the top 20 KEGG pathways between each group were analyzed, and the pathways related to phenolic acid metabolism were selected, as shown in Fig. 7. The C vs. CC analysis revealed significant enrichment of the ABC transporter as well as glycine, serine, and threonine metabolism, cysteine and methionine metabolism, and glycerophospholipid metabolism in the CC treatment group. The ABC transporter pathway of the ABC transporter proteins has a complex cellular detoxification mechanism that helps defend against a variety of heterologous compounds or toxic substances inside and outside the cell. Amino acid pathways in plants improve adaptation to environmental stress. The CC vs. CT analysis showed significant enrichment in the CT treatment for starch and sucrose metabolism, isoflavone biosynthesis, ABC transporter, galactose metabolism, aminosugar and nucleotide sugar metabolism, glycerophospholipid metabolism, and interconversion of pentose and glucuronide. Inoculation with *F. mosseae* improved the metabolic pathways, such as starch and sucrose metabolism and isoflavone biosynthesis, and mitigated the effects of phenolic acids produced by continuous monocropping on the growth of soybeans through the production of sugars, isoflavones, and other substances.

### Correlation analysis of functional genes and metabolites of phenolic acids in soybean roots

**Fig. 8:**
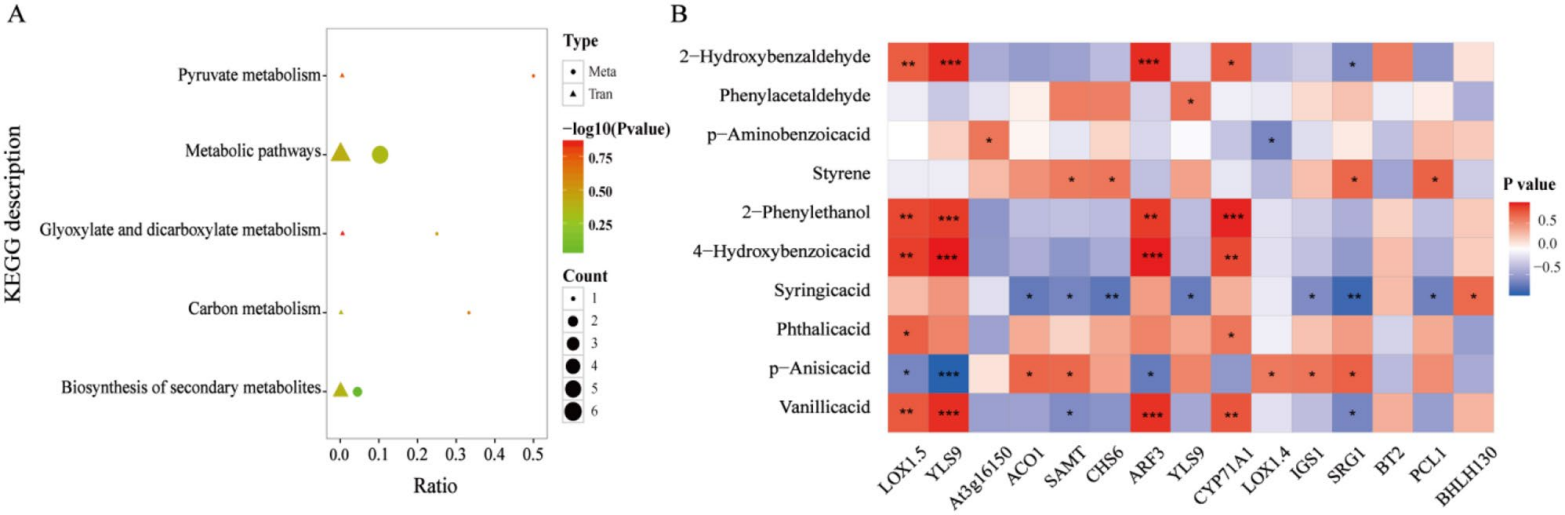
Correlation analysis of the target DEGs and phenolic acid DAMs. **A**, Bubble diagram of the metabolic pathways co-annotated by the DEGs and DAMs in the four treatment groups of C, CC, T, and CT. **B**, heatmap of the correlations between the target DEGs and 10 selected phenolic acid metabolites (*p* < 0.05). (*Figure note*: Triangles represent transcriptome differential gene metabolic pathways and circles represent metabolomic differential metabolic pathways. Graph size indicates the number of numbers and graph color represents the − log_10_(*p*) values.)

A pathway-based approach combining metabolomics and transcriptomics analyses was used to better understand the relationship between the DEGs and the differentially expressed metabolites. Further analysis of the metabolic pathways co-annotated by DEGs and DAMs yielded five co-annotated pathways (Fig. 8A), and these DEGs and differentially expressed metabolites were co-enriched in pyruvate metabolism, glyoxylate and dicarboxylate metabolism, and carbon metabolism. Pyruvate metabolism is closely related to adaptation to stress. Under environmental stress, plants regulate the pyruvate pathway to maintain the balance of energy metabolism and increase resistance to stress. The sugar metabolic pathway is regulated through sugar synthesis and degradation to adapt to the changing needs of the environment.

As phenolic acids are allelochemicals that contribute to autotoxicity in the root system of continuously cropped soybeans, a heatmap of the correlations between 10 phenolic acid metabolites and the DEGs were analyzed (Fig. 8B). As shown in Figs. 2 and 8b-hydroxybenzaldehyde was significantly and positively correlated (*p* < 0.05) with the *YLS9* and *ARF3* genes. 4-Hydroxybenzoic acid, 2-phenylethanol, and vanillic acid were significantly (*p* < 0.05) correlated with the *LOX1.5*, *YLS9*, *ARF3*, and *CYP71A1* genes. Syringic acid was significantly negatively correlated with the *CHS6* and *SRG1* genes (*p* < 0.05). p-Anisic acid was significantly negatively correlated with the *YLS9* gene (*p* < 0.05). These results indicate that continuous cropping can regulate the expression of phenolic acid-producing genes in the soybean root system, which in turn produces phenolic acids that are toxic to soybeans through the regulation of metabolic pathways; whereas inoculation with AMF inhibits the expression of genes related to phenolic acid production in the soybean root system, which in turn reduces enrichment in the relevant metabolic pathways, thereby reducing phenolic acid production in the root system, and ultimately slows down the toxic effects of phenolic acids on soybean.

### Correlation analysis between phenolic acids and soybean plant growth indicators

**Fig. 9:**
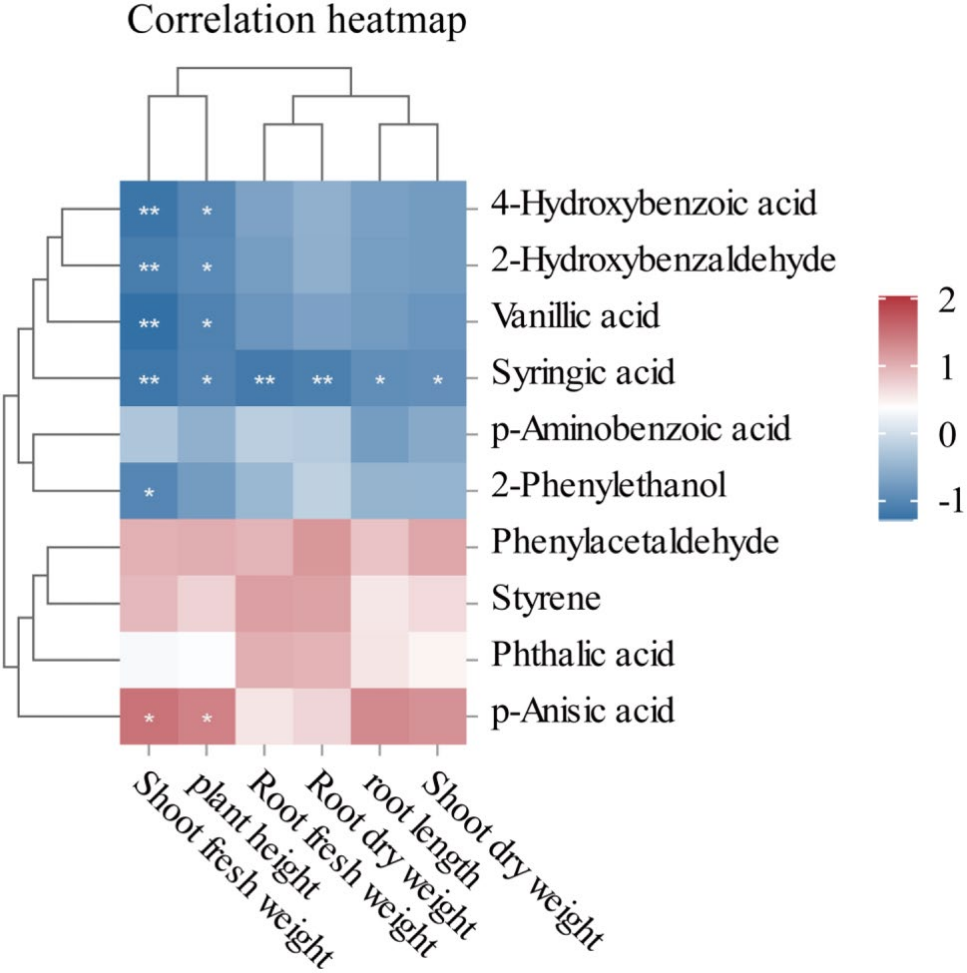
Correlation analysis between phenolic acids and biological indices of the soybean plants. (*Figure note*: Blue boxes indicate a negative correlation, red boxes indicate a positive correlation, darker color indicates a stronger correlation; * indicates a significant correlation (*p* < 0.05),** indicates a highly significant correlation (*p* < 0.01).)

As shown in Fig. 9, the production of phenolic acids was correlated with soybean growth indices, and the height of the plant was negatively correlated (*p* < 0.05) with 4-hydroxybenzoic acid, salicylaldehyde, vanillic acid, and butyric acid. Root length was negatively correlated (*p* < 0.05) with butyric acid. The aboveground fresh weight of the plants was highly significantly negatively correlated (*p* < 0.01) with 4-hydroxybenzoic acid, salicylaldehyde, vanillic acid, butyric acid, and phenylethanol. The belowground fresh weight of the plants was highly significantly negatively correlated (*p* < 0.01) with butyric acid. The aboveground dry weight of the plants was significantly negatively correlated (*p* < 0.05) with butyric acid, and the belowground dry weight of the plants had a highly significant negative correlation (*p* < 0.01) with butyric acid. Phenolic acids produced by continuous monocropping of soybean showed significant negative correlation with soybean growth indexes, and these results indicated that the accumulation of phenolic acids such as 4-hydroxybenzoic acid and vanillic acid produced by continuous monocropping is harmful to plants, affecting the changes of plant height, biomass, and root system of soybean plants, and inhibiting the growth of soybean plants. In contrast, inoculation with AMF reduced the production of phenolic acids in the soybean root system and increased soybean biomass, among other things, which ultimately slowed down the effects of phenolic acids on the growth of soybean plants.

## Discussion

Soybean continuous cropping disorder is a complex problem that has long plagued agricultural production, and the formation and prevention mechanisms have been a hot research topic [24]. Continuous cropping disorders have a significant effect on the growth of soybeans, affecting plant morphological, physiological, biochemical, and molecular processes [25]. Colonization of the soybean root system by AMF increases the area and capacity of the root system to absorb water and mineral elements through a large number of mycelial structures. AMF help regulate plant defense proteins, defense enzymes, and secondary metabolites by affecting host plant-related metabolic pathways and expression of defense genes [26]. Recent advances in genome sequencing, assembly and annotation have facilitated the study of stress tolerance traits in crops. Plants exhibit specific changes in gene expression, metabolism and physiology in response to environmental stress. In this study, we combined transcriptomic and metabolomic correlation analyses to reveal the effects of continuous cropping on changes in gene expression and metabolites regulating phenolic acid production in soybean roots and the mitigating effects of inoculation with *F. mosseae* on phenolic acid toxicity in continuously cropped soybean roots. Continuous cropping negatively affected the growth of soybean plants, by significantly suppressing plant height, root length, and above and belowground fresh and dry weights, which is consistent with Jie et al. [27], and was accompanied by changes in gene expression, metabolic pathways, and metabolites [28].

### AMF regulate gene expression for phenolic acid production in continuous monocropping soybean roots

The use of transcriptomics to analyze the molecular mechanisms of the response of soybeans to phenolic acids has attracted research attention. He et al. [29] used RNA-seq to identify 762 DEGs in the leaves of *Liancuo platycodon grandiflorus*, which were involved in the metabolic pathways of “tyrosine degradation I.”, “Glycogen synthesis”, “Phenylalanine and tyrosine catabolism”, “Signal transduction” and “Immune system”, “Phenylalanine and tyrosine catabolism”, “signal transduction” and “immune system” metabolic pathways. Li et al. [30] identified 6,193 single genes by transcriptome sequencing that were significantly differentially expressed in *Andrographis paniculata* after continuous planting.

In this study, it was observed that continuous monocropping affected the growth and development of soybean plants, with a total of 2,766 DEGs in the C vs. CC group and 1,431 DEGs in the CC vs. CT group. Among the 365 genes selected that were associated with the production of phenolic acids, the expression of genes related to *CHS6*, *PCL1*, *IGS1*, *SAMT*, *SRG*1, *ACO1*, and *YLS9* were significantly higher in the root system of continuously cropped soybeans. Chitin synthase gene (*CHS*) has an important role in plant development and pathogenesis, *CHS6* gene regulates the production of 4-coumarin coenzyme A in the phenylalanine metabolic pathway, and 4-coumarin coenzyme A serves as a synthetic substrate for chalcone synthase, which is known to have a role in regulating the enhancement of disease resistance in soybeans, and in particular *CHS6* is essential for plant pathogenesis [31, 32]. Isoeugenolsynthase (*IGS*) has NADPH-dependent reductase activity [33], and this gene family plays an important role in the response to environmental stress, among others [34]. SA carboxyl methyltransferase (*SAMT*) is a component of the o-hydroxybenzoic acid signaling pathway that catalyzes the conversion of intermediates in the o-hydroxybenzoic acid biosynthetic pathway [35]. *SAMT* is involved in plant defence against pathogens processes and in the catalysis and production of methyl salicylate (MeSA), and its expression is up-regulated during plant expression is up-regulated during pathogen infection [36]. Treatment with benzothiadiazole and SA induces *OsBISAMT1* (Benzothiadiazole-induced *SAMT1* gene) expression in rice leaves, which further improving disease resistance in rice [37]. The *ACO* gene undergoes induced expression by stimuli, such as hormonal signals, external environmental stress, and developmental processes. Genes such as peach *ACO* [38] and tomato *ACO1* [39] are expressed during environmental stress. All of the above gene changes indicate that continuous cropping of soybeans produces growth disorder and induces the expression of genes related to phenolic acids.

The expression of genes, such as *ARF3*, *CYP71A1*, *LOX1.5*, *BHLH130*, and *BT2*, was upregulated after inoculation of soybean roots with AMF in three years of continuous cropping. The *ARF3* gene is involved in the plant’s response to adversity and can regulate the expression of a range of adversity-responsive genes, thereby enhancing the plant’s resistance to adversity [40]. The *CYP71A1* gene plays an important role in plant defence as well as in improving disease resistance, and in rice, the cytochrome P450 family gene *CYP71A1* encodes tryptophan 5-hydroxylase, and the regulation of 5-hydroxytryptophan biosynthesis plays an important role in plant defense and disease resistance [41]. Lipoxygenase (LOX) is the first key enzyme in the jasmonic acid synthetic pathway, and this metabolic pathway produces downstream products jasmonates (JAs) and green leaf volatiles (GLVs), and jasmonic acid in the cells of the plant body can be synthesised through the α-linolenic acid or linoleic acid pathway and other syntheses that are directly involved in the resistance to stress and growth and development [42]. A significant positive correlation has been detected between *Vv LOX2* gene expression and C6 substance content after trauma in grapes, suggesting that this gene plays an important role in the stress response [43]. The *BHLH130* gene plays an important role in plant growth and development and under abiotic stress conditions, and it has now been demonstrated that *bHLH* transcription factors are involved in plant growth and development and disease resistance responses [44]. Sixty-nine *NnbHLH* functions have been predicted in *Nelumbo nucifera*, which are related to developmental physiology and the stress response [45], In addition to its role in growth and development, plants respond to stress by producing a wide range of biologically active and unique metabolites, and *bHLH* plays an important role in the biosynthesis of phenolic acids, which are secondary metabolites of plants [46]. All other upregulated genes had biological functions in the response to stress and toxicity. In conclusion, this study concluded that continuous monocropping leads to toxic effects in the soybean root system and that inoculation with AMF slows down these toxic effects.

### AMF regulate metabolic pathways for the production of phenolic acids by continuous monocropping soybean roots

In this study, the functions of DEGs were further characterized by GO enrichment and KEGG pathway enrichment analyses. GO enrichment analyses of the DEGs revealed enrichment in metabolic processes, cellular processes, single organism processes, catalytic activity, and plasma membrane and organelle pathways. The KEGG pathway enrichment analysis showed that the most highly enriched pathways after inoculation with *F. mosseae* were phenylpropane biosynthesis, starch and sucrose metabolism, and isoflavone biosynthesis metabolism, which are defense-related pathways. Phenylpropane biosynthesis is involved in plant defense metabolism [47]. For example, some phenylpropanoids are important substrates in lignin synthesis, which plays a key role as a protectant, a plant antitoxin, and an antioxidant in higher plants [48]. This is in line with the study of Xing et al. [49] on potato succession. In the present study inoculation with *F. mosseae* significantly enriched the phenylpropanoid biosynthetic pathway, suggesting that inoculation with *F. mosseae* improves the resistance to toxins and inhibits the effects of phenolic acid autotoxins produced by continuously cropped soybean.

### AMF regulate phenolic acid production in the root system of continuous monocropping soybeans

In the present study, it was found that continuous monocropping increased the phenolic acid metabolites in the soybean root system, mainly 4-hydroxybenzoic acid, butyric acid, p-methoxybenzoic acid, phthalic acid, and vanillic acid, and the increase in the content of these metabolites was detrimental to the growth of soybean. Previous studies have shown that phenolic acids induce toxicity, affect soil enzyme activities, nutrient cycling, and ion uptake, and inhibit plant growth [50]. Four phenolic acids, such as p-hydroxybenzoic acid, ferulic acid, cinnamic acid, and p-coumaric acid, steadily accumulated in five strawberry samples with different durations of continuous cropping [8]. Li et al. [51] reported that the root secretion phthalic acid has an inhibitory effect on soybean growth and development. Notably, nine phenolic acids (vanillic acid, vanillin, salicylic acid, protocatechuic acid, butyric acid, benzoic acid, coumaric acid, p-hydroxybenzoic acid, and phthalic acid), as well as ferulic acid and cinnamic acid impede growth of the ginseng embryonic root, and this inhibitory effect increases with the concentration of phenolic acids, suggesting that phenolic acids are a direct impediment to ginseng growth [52]. Therefore, these phenolic acids found in this study were autotoxic substances produced by the continuous root system of soybean, and the content of these phenolic acid metabolites was reduced by inoculation with *F. mosseae*, as shown in the study of Yang et al. [53], where the application of biological agents promoted the degradation of salicylic acid in paeonies and reduced its content. This also proves that AMF can be used as biological agents to reduce the content of phenolic acids.

The metabolome is the biochemical basis of the plant phenotype, and remodeling of the metabolome under stress largely reflects the response and the defense of the plant against stress. Plant phenolic acids are synthesized through the phenylpropane and tyrosine pathways of shikimic acid [54]. There are three main pathways for the manufacture of metabolic intermediates in plants, including the pentose phosphate, starch, and sucrose metabolism, and the interconversion of pentose and glucuronic acid, which provide erythrose-4-phosphate as a precursor for the synthesis of shikimic acid. Based on a KEGG analysis, continuous monocropping and inoculation with *F. mosseae* increased activity in the metabolic pathways involved in starch and sucrose metabolism, isoflavone biosynthesis, and interconversion of pentose and glucuronic acid. Starch and sucrose are essential for plant growth and development and response to abiotic stress [55]. The sucrose and starch synthetic pathways, as the main pathways for energy production and accumulation in plants, maintain activities. In contrast, continuous monocropping leads to insufficient synthesis of energy substances and reduces resistance to external pests and disease invasion.

## Conclusions

**Fig. 10:**
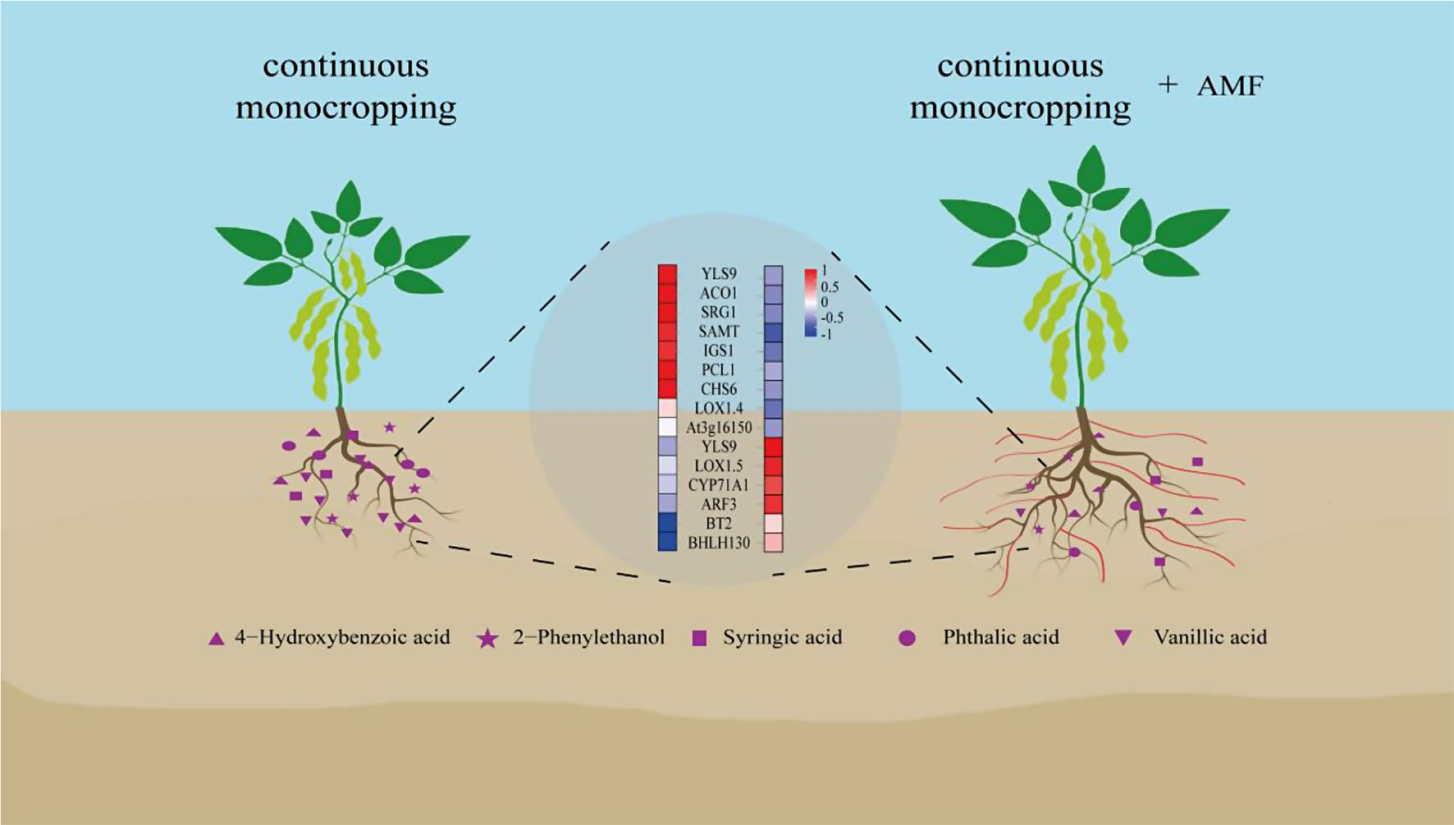
Molecular mechanism of inhibition of phenolic acid autotoxic substances produced by continuous monocropping soybean roots by AMF

In this study, the soybean phenotyping test showed that continuous monocropping inhibited the growth of soybeans, and the inhibition was alleviated by inoculation of *F. mosseae*. Continuous monocropping changed the functional genes, metabolic pathways and metabolites of phenolic acids in soybean roots. After inoculation with *F. mosseae*, the gene expression of phenolic acids in soybean roots was down-regulated, and the metabolic pathways such as phenylpropane biosynthesis in soybean roots were down-regulated, which reduced the production of phenolic acids such as 4-hydroxybenzoic acid and phthalic acid, and improved the tolerance of soybean to phenolic acids produced by continuous cropping. The continuous monocropping obstacle of soybean has been reduced (Fig. 10). This study revealed the molecular mechanism that *F. mosseae* inhibited the production of phenolic acids from soybean roots in continuous monocropping, which provided a new insight for the defense strategy of soybean continuous monocropping, and also provided a theoretical basis for the development and application of AMF biological agents.

## Data Availability

The RNA-seq data obtained in the present study were deposited in the NCBI Sequence Read Archive (SRA, https://www.ncbi. nlm.nih.gov/sra/) under the accession number SRP312573.

## Abbreviations

UPLC: Ultra-Performance Liquid Chromatography
MS/MS: Tandem Mass Spectrometry
DEGs: Differentially Expressed Genes
GO: Gene Ontology
KEGG: Kyoto Encyclopedia Of Genes And Genomics
BP: Biological Process
MF: Molecular Function
CC: Cellular Component
